# Two-Dimensional Automatic Measurement for Nozzle Flow Distribution Using Improved Ultrasonic Sensor

**DOI:** 10.3390/s151026353

**Published:** 2015-10-16

**Authors:** Changyuan Zhai, Chunjiang Zhao, Xiu Wang, Ning Wang, Wei Zou, Wei Li

**Affiliations:** 1 College of Mechanical and Electronic Engineering, Northwest A&F University, Yangling 712100, China; E-Mails: zhaichangyuan@ nwsuaf.edu.cn (C.Z.); liweizibo@nwsuaf.edu.cn (W.L.); 2 National Engineering Research Centre for Information Technology in Agriculture, Beijing 100097, China; E-Mails: wangx@nercita.org.cn (X.W.); zouw@nercita.org.cn (W.Z.); 3 Department of Biosystems and Agricultural Engineering, Oklahoma State University, Stillwater, OK 75078, USA; E-Mail: ning.wang@okstate.edu

**Keywords:** nozzle flow distribution, spray deposition measurement, two-dimensional automatic measurement, ultrasonic sensor

## Abstract

Spray deposition and distribution are affected by many factors, one of which is nozzle flow distribution. A two-dimensional automatic measurement system, which consisted of a conveying unit, a system control unit, an ultrasonic sensor, and a deposition collecting dish, was designed and developed. The system could precisely move an ultrasonic sensor above a pesticide deposition collecting dish to measure the nozzle flow distribution. A sensor sleeve with a PVC tube was designed for the ultrasonic sensor to limit its beam angle in order to measure the liquid level in the small troughs. System performance tests were conducted to verify the designed functions and measurement accuracy. A commercial spray nozzle was also used to measure its flow distribution. The test results showed that the relative error on volume measurement was less than 7.27% when the liquid volume was 2 mL in trough, while the error was less than 4.52% when the liquid volume was 4 mL or more. The developed system was also used to evaluate the flow distribution of a commercial nozzle. It was able to provide the shape and the spraying width of the flow distribution accurately.

## 1. Introduction

Pesticides are widely used in agricultural production for pests and diseases control. However, over-spraying or spray deposition out of targets often cause pesticide residues, which are harmful to human health, environment, especially surface water [1,2]. These impacts have been discovered and studied recently [3,4,5]. Pesticide residues are dependent on initial spray deposit, physical decay due to weather conditions, and plant absorption [6]. Using precision spraying technology to control spray deposit has been recognized as an important approach to reduce pesticide residues [7,8].

Spray deposition and distribution is affected not only by total spray flow rate coming out of a nozzle, but also by many factors, such as nozzle flow distribution, spray direction, air assistance, droplet dynamics [9,10,11]. Careful evaluation of the flow distribution of a nozzle can ensure of the precision of spray. Recently, devices for one-dimensional nozzle flow distribution measurement were designed and used. Fan nozzle flow distribution was measured using a spray sample table, including a groove patternator with some V-shape liquid collecting troughs and measuring cups. The V-shape troughs collected spray liquid from a segment of the spray pattern, and the volume of spray liquid was measured manually or automatically with sensors [12,13,14]. The groove patternator can measure flow distribution of nozzles with one-dimensional shape deposition, however it cannot be used for two-dimensional spray deposition measurement.

Two-dimensional flow distribution measurement is necessary for nozzles with two-dimensional shape spray deposition on a flat field, such as cone shape nozzles. The volume of the spray liquid can be calculated through measuring liquid level when the geometry of a liquid container is fixed and known [15]. Many liquid level sensors have been developed and commercialized. They are based upon hydrostatic pressure sensors [16,17,18], image sensors [19], optical fiber sensors [20,21], capacitance sensors [22,23], microwave sensors [24], ultrasonic sensors, *etc.* Bukhari S.F.A. and Yang W presented a method that allowed the detection of changes in liquid level at micron level using a lead zirconate titanate (PZT) actuated millimeter-sized cantilever [16]. The sensor was a composite structure of two layers: PZT and stainless steel of a few millimeters in length [18]. An imaged-based measurement system using a single digital camera and a circular float to measure fill levels in liquid tanks was presented in [17]. Based on an established relationship between the pixel counts of the diameter of the float in an image and the camera distance, the system effectively measured the liquid level based on the captured images. Design and construction of an optical fiber sensor, which operated based on light intensity modulation, for liquid level detection were reported. The modulated intensity was measured using a pair of fibers for transmitting and receiving light, and a glass prism providing the total and partial reflections [21]. A microwave level sensor for molten glass operating in an industrial furnace was proposed and tested under operative conditions [24]. However, the hydrostatic pressure sensor is intrusive sensor; the image sensor, optical fiber sensor, and capacitance sensor need auxiliary devices put on the liquid or fixed to the container to measure the liquid level; and the microwave sensor is expensive and need complex data processing. They are not suitable for liquid level rapid multiple measurements in different containers.

An ultrasonic sensor is often used in distance measurement. It sends out an ultrasonic signal and receives a signal reflected from a targeted object. The time lapse between sending and receiving the signal and the known sound speed are then used to calculate the distance between the sensor and the targeted object. Because of its low cost, high precision, and non-intrusive measurement, ultrasonic sensors were widely used in liquid level measurement [25,26]. Early in 1997, ultrasonic distance sensors were adopted to measure the liquid levels in bottles on an industrial processing line. This simple ultrasonic distance sensor tested in an industrial plant had a good accuracy and consistency, low cost and high reliability [27]. Recently, a measurement system based on an ultrasonic sensor to accurately determine liquid levels in dynamic environments was experimented and verified on a fuel tank of a running vehicle [28]. Although there are many ultrasonic liquid level sensors available on the market, the sensors are still hard to find, which are required to be with high resolution and be used to sense in a small container in order to evaluate nozzle flow distribution. The beam angle of many available ultrasonic sensors are too big to the container used to evaluate nozzle flow distribution, which often sense the container’s wall rather than the liquid inside.

This paper presents the development of a two-dimensional, automatic liquid level measurement system for nozzle flow distribution using an ultrasonic sensor and a pesticide deposition collecting dish with small troughs.

This paper is organized as follows: Section 2 presents the design of a two-dimensional, automatic liquid level measurement system, the optimization of an ultrasonic sensor, and the development of computer software. Section 3 reports experiments and results. Section 4 draws conclusions.

## 2. Materials and Methods

### 2.1. Design of a Two-Dimensional Liquid-Level Measurement System

**Figure 1:**
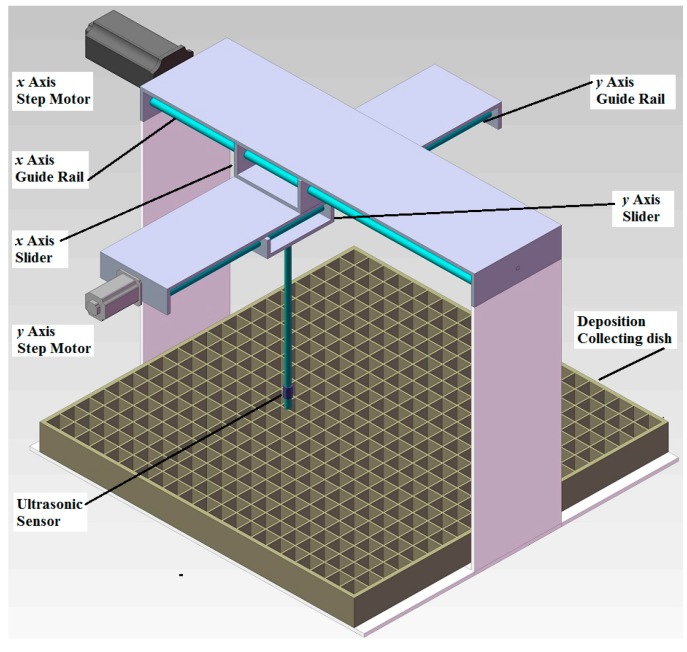
Structural diagram of the two-dimensional liquid-level measurement system for evaluating nozzle flow distribution.

A two-dimensional liquid-level measurement system was designed and developed (Figure 1), which could precisely move an ultrasonic sensor above a pesticide deposition collecting dish to measure the nozzle flow distribution. The system consisted of a conveying unit, a system control unit, an ultrasonic sensor, and a deposition collecting dish. The conveying unit included an *x*-axis slider and a *y*-axis slider, which was driven by a step motor and a guide rail, respectively. The *y*-axis slider was fixed on the *x-*axis slider. The ultrasonic sensor was amounted on the *y*-axis slider. The step motor could drive the sliders precisely along the guide rail with a precision of 0.04 mm. The maximum ranges of *x-* and *y*-axes were 0.600 m, respectively. A square deposition collecting dish was designed and machined with the side length of 0.470 m and a height of 0.040 m. It had 21 × 21 evenly arranged, square collecting troughs. Each trough was with a side length of 20 mm and a depth of 35 mm, which could hold 0.014 L liquid.

### 2.2. Design of the System Control Unit

**Figure 2:**
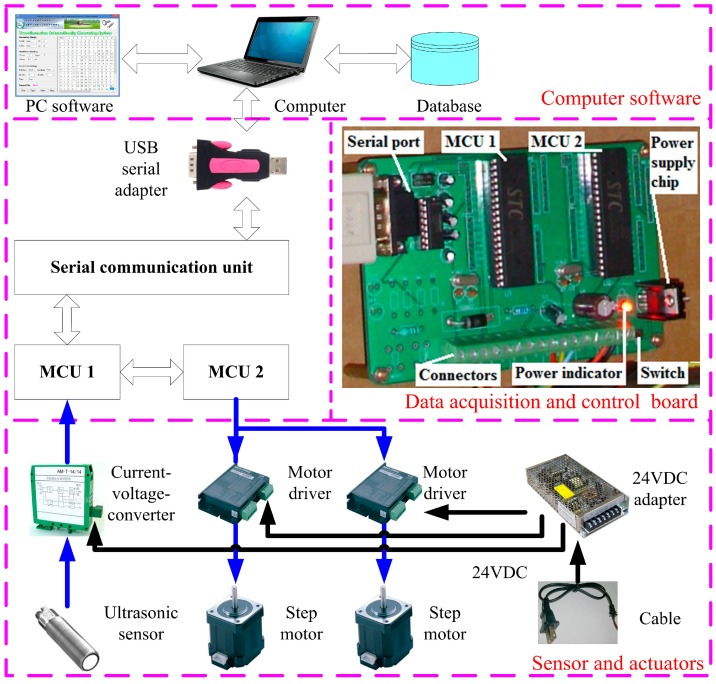
General block diagram of the system control unit.

The system control unit was used to control the movements of the ultrasonic sensor to a specific location, read sensor data, calculate and record the volume of the pesticide in each trough, and show the results on a computer screen. The system control unit contained a module of sensor and actuators, a module of data acquisition and control board, and a computer software module (Figure 2). The module of sensor and actuators included an ultrasonic sensor (946-A4V-2D-2C0-380E, Honeywell International Inc., Morristown, NJ, USA), a current voltage converter (AM-T-I4/U5, Le Qing Teng Er Electric Co., Ltd., Leqing, China), two step motors (34HS300B, Beijing Flourishing Start Digital Technology Co., Ltd., Beijing, China), two motor drivers (SH-2H090M, Beijing Flourishing Start Digital Technology Co., Ltd., Beijing, China) and a 24 V DC adapter. The ultrasonic sensor could measure a distance range of 30–500 mm with a beam angle of 5°. It was powered by 24 V DC and output a current signal of 4–20 mA. A current-voltage-converter was used to convert the current signal of the ultrasonic sensor into a voltage signal to be read by a microcontroller. The motor drivers were used to receive control signals from a microcontroller and control the movements of the two step motors. The power adapter supplied 24 V DC power to the current voltage converter and the two motor drivers.

The data acquisition and control module included two microcontrollers (MCU), a serial communication unit, and a USB serial adapter. The MCU1 was used to read the signal from the ultrasonic sensor, while the MCU2 was used to control the step motors. This module could also communicate with a computer through the serial communication unit and the USB serial adapter. Computer software was developed to allow a PC to send commands to the data acquisition and control module to move the sensor by the two step motors, to receive and analyze the signals from the ultrasonic sensor, and to record and display the results on the screen.

### 2.3. Enclosure of the Ultrasonic Sensor

**Figure 3:**
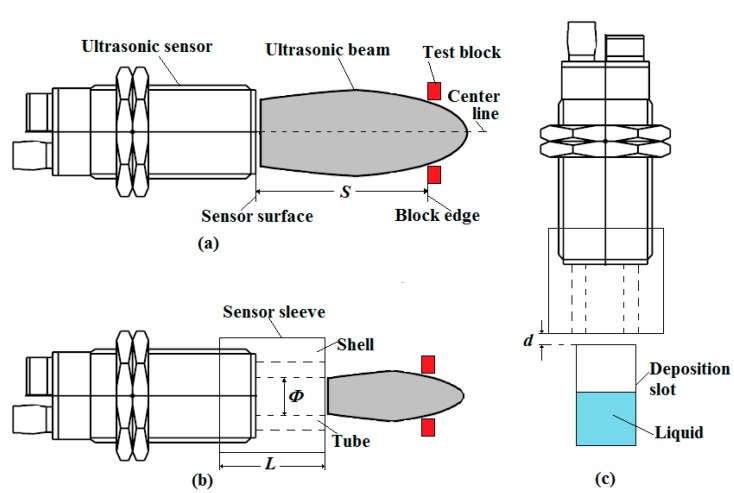
Beam diameter test of the ultrasonic sensor and schematic of deposition measuring. (**a**) Beam diameter test of the sensor without sleeve; (**b**) Beam diameter test of the sensor with sleeve; (**c**) Schematic of deposition measuring using the sensor with sleeve.

The beam angle (or diameter) of the ultrasonic sensor was very important for the developed system. It should be smaller than the side length of the trough on the deposition collecting dish. Otherwise, the sensor would detect the wall of the trough rather than the surface of the pesticide liquid. The beam diameter was measured using two blocks. They were aligned in parallel with the surface of the ultrasonic sensor (Figure 3a). The distance (*S*) between the edge of the two blocks and the sensor surface was set from 50 mm to 130 mm with an incremental step of 20 mm. When the distance was less than 50 mm, the signal of the ultrasonic sensor was not stable due to an inherited dead zone. At each distance *S*, both blocks were moved toward the beam center line until they could be detected. Then the range between the two blocks, which was the diameter of the ultrasonic beam, was measured. The initial test results showed that the beam diameter was always larger than the side length of the trough (20 mm). The sensor could not directly measure the level of the pesticide liquid in the trough on the deposition collecting dish.

In order to make the diameter of ultrasound beam smaller, a sensor sleeve was designed using an aluminum shell with a PVC tube in it (Figure 3b,c). The PVC tube reduced the beam angle of the ultrasound sensor. However, if the PVC tube was too long or the inner diameter of the tube was too small, the ultrasound might not be able to go through the tube. The signal received by the ultrasonic sensor could be that reflected from the wall of the tube. Hence, the length selection of the PVC tube was very critical. An experiment was conducted to select the best size of the PVC tube. Two tubes with different inner diameters were cut into sections with different lengths (Table 1).

**Table 1:** Test to find an available size of the tube in the sleeve.

Tube Inner Diameter (mm)	Was the Wall of the Tube Detected?				
12.0	Tube length (mm)	113	91	72	55
	Detected?	Yes	Yes	Yes	Yes
14.5	Tube length (mm)	152	128	107	89
	Detected?	Yes	Yes	Yes	No

The distance signal from the ultrasonic sensor, indicating the liquid level, was converted into a 0–5 V DC voltage signal and acquired by an analog input channel of MCU1. In order to calculate the liquid volume in each trough on the deposition collecting dish, the equation relationship between them was established through a calibration test. Six troughs were randomly chosen, in which a certain amount of water was dropped into manually using a pipettor (Table 2). For each trough, the distance signal of the ultrasonic sensor was acquired five times using the data acquisition and control board. The maximum and minimum readings were removed in each group of the five readings. An average reading was calculated based on the rest three readings. Same approach was used on an empty trough to gain the zero-height-reference. The liquid level was calculated according to Equation (1):

Liquid level in a trough = *d_1_* − *d_0_* (1)

where *d_1_* was the distance measured from the trough and *d_0_* was the zero-height-reference. The fitting curves of the ultrasonic sensor calibration for liquid volume measurement were obtained based on linear fitting, quadratic fitting, cubic fitting and fourth degree polynomial fitting, respectively.

**Table 2:** The ultrasonic sensor calibration for liquid volume measurement.

Liquid Volume/mL	Ultrasonic Sensor Output						Liquid Level Reading
	Reading 1	Reading 2	Reading 3	Reading 4	Reading 5	Average Reading	
0	480	481	479	481	480	480.3	0.0
3	436	435	440	437	435	436.0	44.3
6	388	391	390	388	387	388.7	91.7
9	324	324	324	324	326	324.0	156.3
12	274	273	276	274	272	273.7	206.7
14	248	247	248	249	249	248.3	232.0

### 2.4. Development of Computer Software

Computer software was developed to run on the Microsoft Windows operation system to support the communication between the PC and the data acquisition and control board through an RS232 serial port, to record the nozzle flow distribution into a database, and to interact with the software users. The software included a serial port communication module, an Access database module, and a software interface module.

The serial port module was designed based on C# language using SerialPort class, to receive the distance data from the ultrasonic sensor and send commands to the data acquisition and control board. Both the distance data and the commands were ASCII strings. The serial port settings, such as the port name, the baud rate, the data bits, the stop bits, and the parity, could be set by users as needed. The setting parameters were stored in a XML file.

In order to record all the data, an Access database was established. Two types of tables were setup in the database to store the liquid volume in the troughs, named “TableList”, and other information, named “FileName”, respectively. “FileName” was unique and given by the user to identify each measurement.

**Figure 4:**
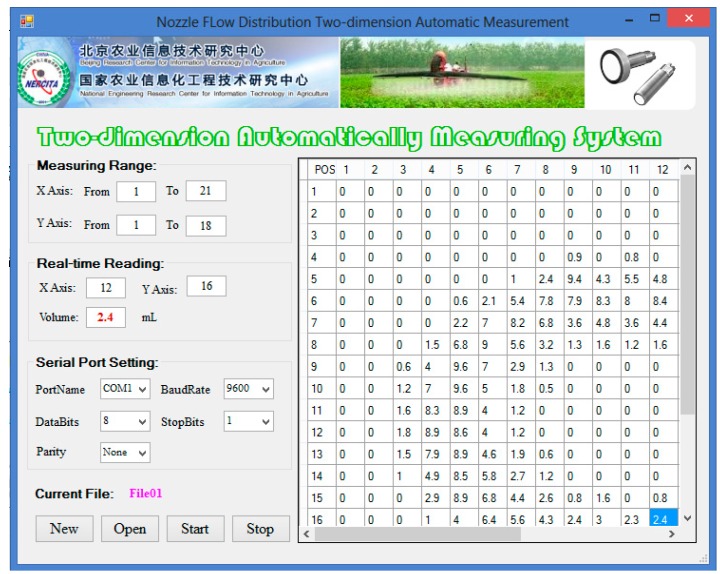
The computer software interface of nozzle flow distribution measuring system.

The software interface module was developed using C# language based on Microsoft Visual Studio 2012 (Figure 4). It displayed the *x*- and *y*-axes measuring ranges, the real-time reading, the serial port setting, the current file name, and all the historical reading data. The software was designed to be user-friendly with the following procedures:

1. Set up the measuring ranges and the serial port.
2. Create a new file by clicking the “New” button, or open an existing file by clicking the “Open” button.
3. Run the software using the “Start” button. The software sent the commands to the data acquisition and control board to move the ultrasonic sensor step by step. When the sensor was above a trough, the sensor data were acquired five times and the average volume was calculated.
4. Stop the software by pressing the “Stop” button.

### 2.5. System Performance Test

To evaluate the accuracy of the developed system, 21 troughs were randomly chosen, in which a certain amount of water was dropped into manually using a pipettor (Table 3). The volume of water ranged from 0 mL to 14 mL with an incremental step of 2 mL. For each trough, the distance signals from the ultrasonic sensor were read and pre-processed by the data acquisition and control board. The liquid level and volume in a trough were calculated. The relative errors between the liquid volumes and the actual liquid volumes were obtained.

### 2.6. Nozzle Flow Distribution Measurement

**Figure 5:**
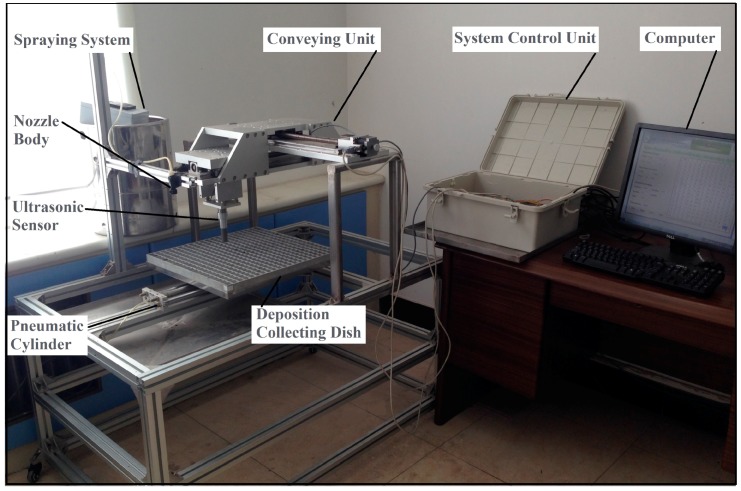
The developed two-dimensional automatic measuring system and a spraying system.

The two-dimensional automatic measuring system was developed and could be used together with a spraying system. The working pressure in the spraying system could be set manually, and the deposition collecting dish could be moved from the spraying system to the two-dimensional measuring system using a pneumatic cylinder after spraying (Figure 5). The two systems were applied for the flow distribution measurement of a commercial nozzle, Teejet AITXA 8004. The nozzle was fixed at a height of 0.20 m above the center of the square deposition collecting dish. Pesticide liquid was sprayed continuously and vertically downward for 20.03 s with a spraying pressure 0.3 MPa. The two-dimensional flow distribution of the nozzle was obtained. The flow distribution horizontal and vertical sum statistics, which were calculated by adding the liquid volume together in the horizontal and vertical directions, respectively, and spraying widths were calculated, which were indispensable for determining spray nozzles layout of an orchard sprayer.

**Table 3:** Precision test of the two-dimensional automatic measuring system.

Real Liquid Volume/mL	Ultrasonic Sensor Reading							Liquid Volume Calculated/mL	Relative Error/%
	Reading 1	Reading 2	Reading 3	Reading 4	Reading 5	Average Reading	Liquid Level Reading		
0	480	478	476	479	479	478.7	0	0	0
2	453	446	449	452	450	450.3	28.4	2.12	6.12
2	449	453	450	451	449	450.0	28.7	2.15	7.27
2	448	451	452	448	451	450.0	28.7	2.15	7.27
4	419	420	418	418	416	418.3	60.4	4.14	3.56
4	417	422	416	420	418	418.3	60.4	4.14	3.56
4	416	418	418	419	417	417.7	61.0	4.18	4.52
6	379	379	379	380	380	379.3	99.4	6.21	3.56
6	380	379	379	378	379	379.0	99.7	6.23	3.84
6	380	377	380	379	380	379.7	99.0	6.20	3.28
8	348	349	346	351	348	348.3	130.4	7.74	−3.23
8	341	342	341	343	340	341.3	137.4	8.09	1.13
8	342	343	344	338	352	343.0	135.7	8.01	0.08
10	305	304	303	304	303	303.7	175.0	10.09	0.90
10	301	306	306	306	307	306.0	172.7	9.96	−0.43
10	308	310	310	311	309	309.7	169.0	9.75	−2.50
12	277	275	276	275	276	275.7	203.0	11.83	−1.43
12	274	272	272	273	273	272.7	206.0	12.03	0.27
12	278	277	274	278	275	276.7	202.0	11.76	−1.99
14	247	247	248	248	249	247.7	231.0	13.91	−0.67
14	247	248	248	247	246	247.3	231.4	13.93	−0.47
14	248	249	247	248	246	247.7	231.0	13.91	−0.67

## 3. Experiments and Results

### 3.1. Beam Diameter Test

The test results in Table 1 showed that the PVC tube in the sensor sleeve with an inner diameter smaller than 12 mm could not be used. The best fit for the sensor sleeve should be with an inner diameter of 14.5 mm and a length of 89 mm.

**Figure 6:**
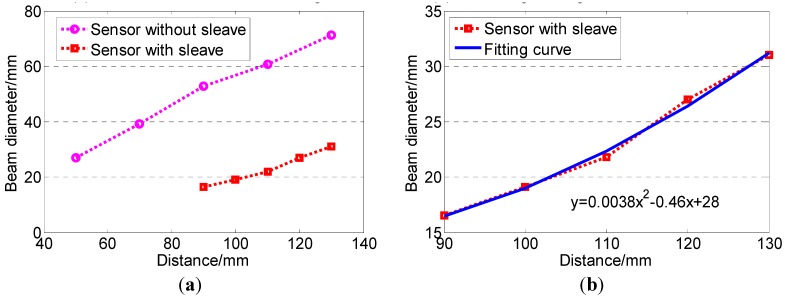
Beam diameter of the ultrasonic sensor changes because of the sleeve. (**a**) Sensor sleeve affects beam angle; (**b**) Beam angle fitting curve of sensor with sleeve.

The beam diameter of the ultrasonic sensor with the sleeve became much smaller than that without a sleeve (Figure 6a). When the distance from the sensor surface was short, the beam diameter was smaller than the side length of the trough (20 mm). The beam angle fitting curve of the sensor with the sleeve was shown in Figure 6b, and the relationship between the distance and beam diameter was shown in Equation (2).

*D* = 0.00379 *l*^2^ − 0.464 *l* + 27.5 (2)

where *l* was the distance away from the ultrasonic sensor surface, in mm; and *D* was the beam diameter of the ultrasonic sensor, in mm.

According the Equation (2), it could be calculated that if the distance was shorter than 103 mm, the beam diameter was shorter than 20 mm. In order to make the beam diameter smaller than the side length of the trough, the distance between the sleeve and deposition trough *d* (Figure 3c) should be less than 14 mm.

### 3.2. Calibration for the Volume Calculation

**Figure 7:**
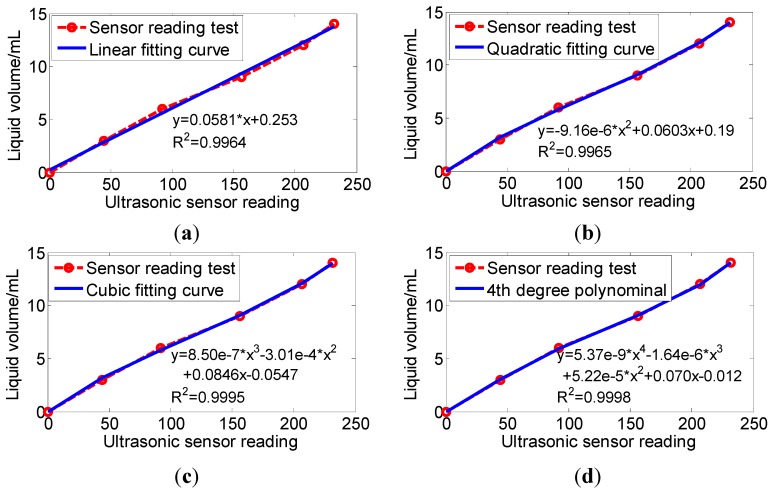
Fitting cure of the ultrasonic sensor calibration for liquid volume measurement. (**a**) Linear fitting curve; (**b**) Quadratic fitting curve; (**c**) Cubic fitting curve; (**d**) 4th degree polynominal fitting curve.

The ultrasonic sensor liquid level reading at different volumes were calculated and shown in Table 2. The fitting curves were obtained between the liquid volume and the ultrasonic sensor liquid level reading (Figure 7). Figure 7 showed that the R^2^ was above 0.999 when the order of the polynomial was equal to or higher than three. Therefore, the cubic fitting cure was chosen (Equation (3)).

*y* = 0.00000085 *x*^3^ − 0.000301 *x*^2^ + 0.0846 *x* − 0.0547 (3)

where *x* was the ultrasonic sensor liquid level reading; and *y* was the liquid volume in a trough, in mL.

According to Equation (3), if the ultrasonic sensor liquid level reading *x* was less than 0.648, the liquid volume *y* would be small and negative. In this circumstance, the actual liquid volume was not more than 0.045 mL, which was so small that the liquid volume *y* could be set to 0 approximately when it was small and negative.

### 3.3. System Performance Test

The relative error between the liquid volumes and the actual liquid volumes were obtained (Table 3). The results showed that the relative error was less than 7.27% when the liquid volume was 2 mL in a trough, while the error was less than 4.52% when the liquid volume was 4 mL or more.

### 3.4. Nozzle Flow Distribution Measurement Application

**Figure 8:**
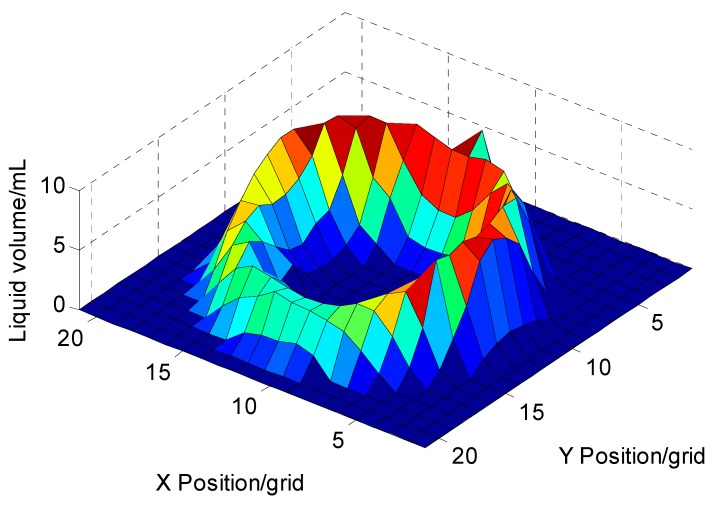
Two-dimensional flow distribution measurement result of a nozzle of Teejet AITXA 8004.

**Figure 9:**
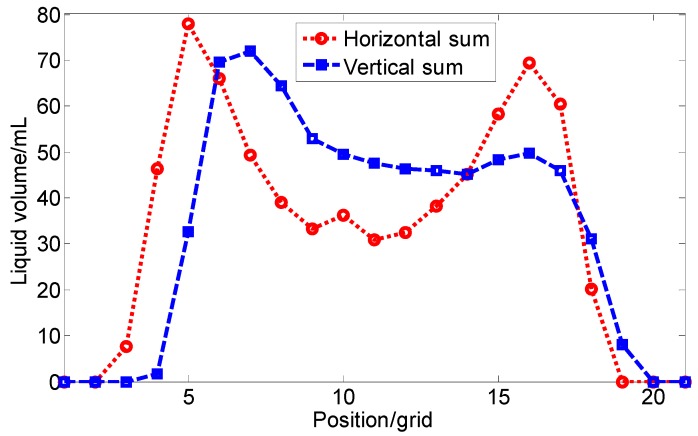
Horizontal and vertical sum statistics of flow distribution of Teejet AITXA 8004 nozzle.

The measured flow distribution for the nozzle, Teejet AITXA 8004 was in a hollow cone-shape (Figure 8). Each grid was relevant to a trough on the square deposition collecting dish. Beneath the center of the nozzle, there was almost no liquid. The horizontal and vertical sum statistical results showed that when the nozzle height was at 0.20 m, the spraying width was about 13 grid (0.26 m) (Figure 9). The curves of horizontal and vertical sum statistics with “double-hump” shape were similar in the spray range.

Flow distributions of nozzles were fundamental information for nozzle arrangement design and spray deposit variable-rate control for an autonomous spaying system, especially a spraying robot. The spacing distance between nozzles on a spray boom needed to be calculated based on horizontal and vertical sum statistics of flow distributions. The spraying robot deposit control was based upon nozzle flow distributions at different spraying pressures. The developed two-dimensional automatic measuring system could significantly improve the measurement accuracy and efficiency to support autonomous spaying systems.

## 4. Conclusions

A two-dimensional liquid-level measurement system was designed and developed that could precisely move an ultrasonic sensor above a pesticide deposition collecting dish to measure the nozzle flow distribution.

A sensor sleeve with a PVC tube was designed for the ultrasonic sensor to measure the liquid level in small troughs whose side length was shorter than the original diameter of the ultrasound beam. The test result showed that the PVC tube with an inner diameter of 14.5 mm and a length of 89 mm was the best fit.

The results of the system test showed that the relative error was less than 7.27% when the liquid volume was 2 mL in a trough, while the error was less than 4.52% when the liquid volume was 4 mL or more.

The two-dimensional automatic measuring system was applied for the flow distribution measurement of a commercial nozzle of Teejet AITXA 8004. The flow distribution was of hollow cone-shape, and its curves of horizontal and vertical sum statistics were of “double-hump” shapes with a spraying width of about 13 grids (0.26 m) when the nozzle height was 0.20 m.
